# Diagnostic and therapeutic precision in cardiovascular diseases in the neonatal intensive care

**DOI:** 10.1038/s41372-025-02317-x

**Published:** 2025-05-10

**Authors:** Arvind Sehgal, Matthew R. Buckingham, Rachael M. Hyland, Patrick J. McNamara

**Affiliations:** 1 Monash Children’s Hospital, Monash University, Melbourne, VIC Australia; 2 Department of Pediatrics, Monash University, Melbourne, VIC Australia; 3 Waitaha Christchurch Neonatal Unit, The Whatu Ora Health, Christchurch, New Zealand; 4 Department of Pediatrics, University of Iowa, Iowa City, IA USA; 5 Division of Neonatology, University of Iowa Stead Family Children’s Hospital, Iowa City, IA USA

**Keywords:** Cardiovascular diseases, Outcomes research

## Abstract

While patent ductus arteriosus, pulmonary hypertension, and systemic hypotension are key medical issues amongst neonates, there are other common biological conditions which present with distinct physiological diagnostic and therapeutic challenges. This review focuses on such hemodynamic considerations in cardiomyopathy accompanying infants of diabetic mothers, twin-to-twin transfusion syndrome, and left heart pathology in infants with severe chronic lung disease. It details the pathophysiological mechanisms, diagnostic approaches, and therapeutic strategies essential for optimizing cardiovascular stability in this fragile cohort. A regimented, protocol-driven approach may lead to an increased risk of unintended treatment side effects in patients where diagnostic precision is low. This review provides a rational basis of the management of hemodynamic instability, at the same time laying out knowledge gaps and considerations for future research.

## Introduction

Neonatal hemodynamic care has traditionally focused on patent ductus arteriosus (PDA), systemic hypotension, and pulmonary hypertension, each of which can significantly impact their clinical outcomes. Oftentimes, a singular approach to maintaining strict blood pressure targets using non-specific vasopressors is adopted with little consideration of the underlying cardiovascular phenotype. An imprecise approach may lead to an increased risk of unintended treatment side effects in patients where diagnostic precision is low. A comprehensive understanding of the unique developmental and physiologic considerations of neonates, the variance in underlying disease phenotypes for similar clinical presentations is essential towards providing optimal neonatal care. In addition, the transition from fetal to neonatal circulation is complex and often precarious in preterm infants due to their immature cardiovascular systems. Several recent review articles highlight the importance of physiology in routine clinical practice and the need for consideration of diverse hemodynamic phenotypes [1, 2]. The delicate balance of maintaining adequate organ perfusion while avoiding excessive fluid overload or pressure-induced injury necessitates a nuanced understanding of neonatal hemodynamics.

There are, however, several additional biological conditions that have distinct developmental and physiological challenges that require specialized hemodynamic management. This article explores the special hemodynamic considerations in infants, highlighting the pathophysiological mechanisms, diagnostic approaches, and therapeutic strategies essential for optimizing cardiovascular stability and supporting overall health in this fragile cohort. Of these, cardiomyopathy in infants of diabetic mothers (IDM) is the most common, with an incidence rate ranging from 13% to 44% presents a significant clinical challenge [3]. The severity of this condition, which correlates with maternal glycaemic control during pregnancy and the consequential hyperinsulinemic environment, can result in diverse cardiac phenotypes; specifically, the hemodynamic burden, which can lead to hypertrophic cardiomyopathy (HCM) and associated complications such as low cardiac output and pulmonary hypertension [4]. The variability in the clinical presentation of HCM among these infants underscores the need for individualized hemodynamic assessment to guide therapeutic strategies effectively. This review aims to synthesize current knowledge on the cardiac implications of IDM, drawing parallels with related syndromes like twin-to-twin transfusion (TTTS) and bronchopulmonary dysplasia (BPD), to underscore the need for comprehensive neonatal cardiac assessment and tailored management strategies. Recognizing and addressing these hemodynamic factors through targeted echocardiography assessments and interventions, such as afterload reduction, are essential in mitigating the adverse impact on the cardiorespiratory system. Accurate hemodynamic assessment and management are therefore critical in addressing the complex cardiovascular challenges faced by infants with conditions such as cardiomyopathy in IDM, TTTS, and BPD. The recent guidelines for targeted neonatal echocardiography (TNE) highlight the unique aspects of these conditions, the limitations of routine clinical surveillance, and provide recommendations for mandatory TNE screening [5].

## Cardiomyopathy in infants of diabetic mothers (IDM)

**Fig. 1:**
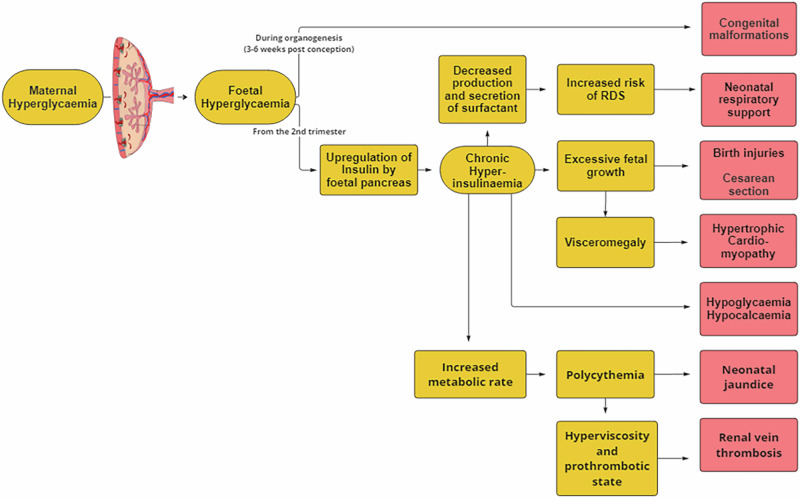
Fetal-neonatal cascade of effects in maternal diabetes (Adapted from Kallem 2020).

Infants are exposed to hyperglycemia during pregnancy, and the resulting fetal hyperglycemia and upregulation of insulin production by the fetal pancreas are responsible for excessive fetal growth. This results in several different cardiac phenotypes including congenital heart disease, cardiac muscle hypertrophy, and cardiac and respiratory maladaptation after birth. Figure 1 illustrates the cascade of events that lead to neonatal complications through the effect of a sustained hyperinsulinemic environment *in utero*.

### Case 1

**Fig. 2:**
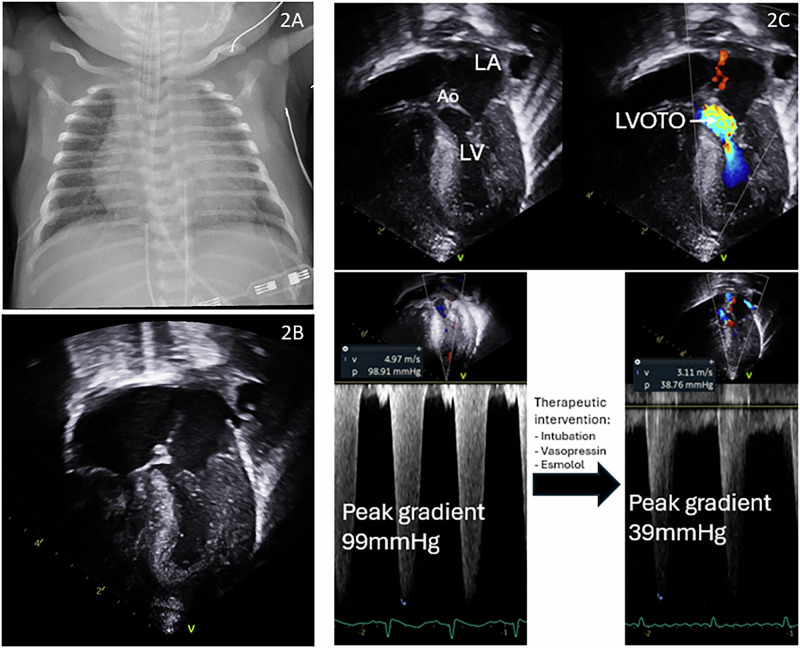
Initial and subsequent imaging showing resolution of cardiac hypertrophy. **A** Initial chest X-ray showing cardiomegaly. **B** 2-dimensional echocardiogram showing asymmetrical septal hypertrophy. **C** Selected echocardiography clips with LVOT gradient pre and post therapeutic interventions. (LA: left atrium; Ao: Aorta; LV: left ventricle; LVOTO: LV outflow tract obstruction).

A male infant was born at term gestation, and pregnancy was complicated by maternal uncontrolled type 2 diabetes mellitus (on insulin therapy), obesity, anemia, and fetal macrosomia. He was delivered by cesarean due to a non-reassuring fetal status. Screening TNE was requested by the clinical team due to antenatal risk factors, raised serum lactate, cardiomegaly on CXR, and respiratory failure requiring continuous positive airway pressure ventilation. This noted significant asymmetric left ventricular (LV) hypertrophy (septal predominance) (LV posterior wall in diastole z-score 4.6, interventricular septum dimeter in diastole z-score 2.7) with mildly decreased LV function (ejection fraction 54% by Simpsons biplane, global strain-10.4) and evidence of dynamic LV outflow tract obstruction (peak gradient of 95 mmHg across the LV outflow tract), systemic level pulmonary pressures based on bidirectional flow through the PDA (68% and 69% left to right by time and by velocity time integral respectively), and a moderate sized atrial communication with bidirectional flow. He was subsequently intubated to support LV function, and started on vasopressin at 1 mU/kg/min to promote LV filling (targeting 50th percentile blood pressure for age), and sedated. Echocardiographic parameters subsequently improved with these interventions and the LV outflow tract gradient dropped to the low 50’s, however, on postnatal day 4 he developed spells of desaturation (Fig. 2). Repeat assessment showed worsened LV outflow tract obstruction and he was additionally started on Esmolol infusion titrated to maintain heart rates <120 beats/min. His gradient again responded favorably, down to the 30s, and he was weaned off of vasopressin. Over the course of the next several weeks, he was successfully extubated, and Esmolol was transitioned to propranolol, which was weaned off prior to discharge. Echocardiogram prior to discharge had no significant LV outflow tract obstruction but continued septal hypertrophy, which resolved during follow-up. Table 1 summarizes the clinical features.

**Table 1:** Summary of clinical presentations of the three cases.

Clinical scenario	GA (weeks)	Birth weight	Antecedent therapies	Concurrent medical issues	Investigation	Therapy	Outcome
IDM with asymmetric HCM	39	4075 g	*Hypoglycemic treated with dextrose bolus -RDS requiring CPAP	*Macrosomia	*CXR *Serial TNE *Serial assessment of lactate *Co-management with pediatric cardiology	-*Intubation to increase transmural pressure gradient and support LV function *Vasopressin to promote LV filling *Rate control with esmolol, and sedation	*Improved LVOT gradient *Discharge home in room air
TTTS-recipient twin	28	1290 g	*No fetal intervention performed *Intubated at delivery, Apgar scores 4, 8. *Surfactant for RDS	*RDS secondary to prematurity	Serial TNE to guide therapies	*Dobutamine *Epinephrine	-Off dobutamine day 2, off epinephrine day 5, with normal cardiac function and initially persistent hypertrophy. -TNE prior to discharge with normal biventricular size and function, no evidence of pulmonary hypertension. -Discharged home at PMA 46 weeks (130 days) on low flow oxygen
BPD associated cPH	27	560 g	*Intubation *Surfactant for RDS *Paracetamol for PDA closure	Between 27 and 36 weeks: *Inability to extubate *Need for 35–60% oxygen *Unexplained splinting accompanied by increased FiO_2_ *Systemic hypertension	TNE to exclude pulmonary artery hypertension	*Co-management with pediatric cardiology *Captopril for 5 weeks	*Cessation of splinting episodes *Successful extubation *Improved cardiac indices^ *Discharge home on low flow oxygen and maintenance captopril

*BPD* bronchopulmonary dysplasia, *cPH* chronic pulmonary hypertension, *FiO*_*2*_ fractional inspired oxygen. ^ (Right ventricular output [ml/kg/min] from 142 to 204 and left ventricular output [ml/kg/min] from 181 to 240), *TNE* targeted neonatal echocardiography, *IDM* infant of diabetic mother, *HCM* hypertrophic cardiomyopathy, *CXR* chest radiograph, *GA* gestational age, *PDA* patent ductus arteriosus, *RDS* respiratory distress syndrome, *TTTS* twin to twin transfusion syndrome, *PMA* postmenstrual age, *CPAP* continous positive airway pressure, *LVOT* left ventricular outflow tract.

#### Discussion

**Fig. 3:**
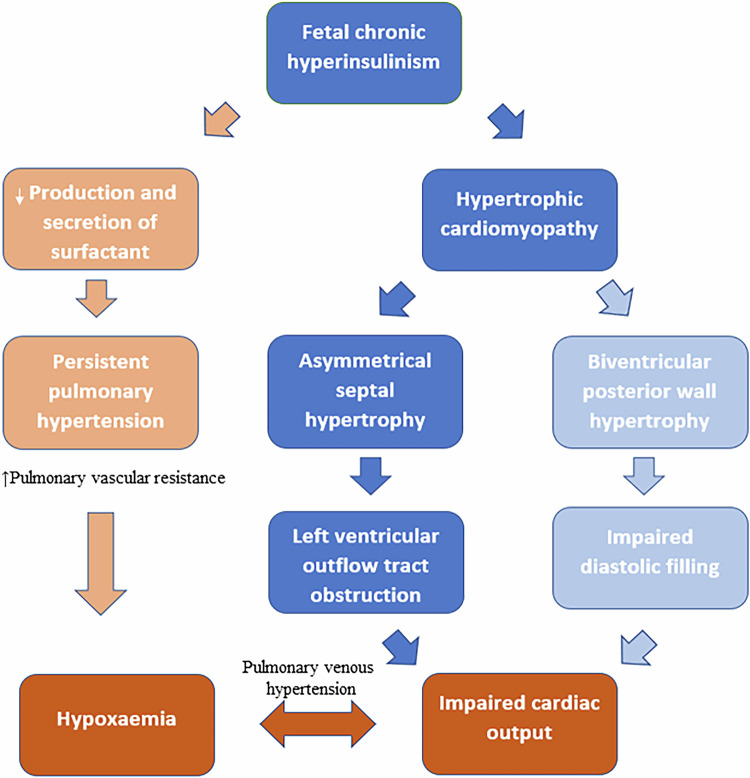
Dynamic effects of fetal hyperinsulinism on infant circulation and oxygenation.

Fetal myocardial hypertrophy has been reported in up to 30% of antenatal scans of pregnancies complicated by maternal diabetes [6]. Histologically, hyperplastic cardiomyocytes with a normal configuration (rather than disarray of muscle fibers, intramyocardial fibrosis, or interstitial collagen depositions such as those seen in other intrinsic diseases of the heart) are noted. Putatively, this might explain why the typically asymmetrical septal hypertrophy is reversible and less prone to intractable arrhythmias. Severe basal septal thickness may lead to apposition of the anterior leaflet of the mitral valve to the interventricular septum during systole and obstruct the LV outflow tract. In some cases, it may also affect the right and left posterior walls symmetrically [7]. These changes can manifest as impaired ventricular relaxation and diastolic filling, which ultimately lead to poor cardiac output. It is plausible that some patients may develop a post-capillary phenotype, which may lead to impaired oxygenation and pulmonary venous hypertension. Fetal hyperinsulinism can also cause early elevated pulmonary pressure and pulmonary vascular resistance, thus exacerbating the infant’s hypoxemic state [8]. Figure 3 depicts the dynamic effects of fetal hyperinsulinism on systemic hemodynamics and oxygenation. Recent consensus recommendations advocate that all IDMs with clinical signs of low cardiac output or  pulmonary hypertension (PH) (such as cyanosis or tachypnoea and cardiomegaly on chest X-ray) should have a comprehensive echocardiography assessment [5]. This will allow for the evaluation of any dynamic obstruction to the LV outflow tract, presence and severity of diastolic and systolic dysfunction, and impact on pulmonary circulation, thereby facilitating the need for physiology-driven interventions. Since the cohort is at a higher risk of congenital heart disease, any suspicion should trigger a request for evaluation by a pediatric cardiologist. Key measurements, principles of assessment, and probe position/view required have been summarized recently [5].

The clinical management of infants with HCM and a low cardiac output state remains quite variable. A recent review highlighted the heterogeneity in therapeutic interventions [9]. A retrospective study (from 2004 to 2019) of hospitalized IDM patients in their first two postnatal weeks noted that IDM related HCM was associated with greater in-hospital mortality (4.9 vs. 1.3%, *p* < 0.001), longer duration of stay, need for (and longer duration of) mechanical ventilation, and adjusted total cost of care. Multivariable logistic regression to assess the association between HCM and mortality noted that other medical conditions independently associated with mortality were PH and prematurity. Inotropes and vasopressors were prescribed in nearly half of infants with HCM, inhaled nitric oxide in 40%, diuretics in 35%, and β-blockers such as Esmolol and propranolol in 17% of patients. Although supportive care with careful management of hypovolaemia and potential PH remains the priority, β-blockers are used for managing cardiac stiffness and decreased diastolic filling. Guiding physiologic principles for acute hemodynamic care include the avoidance of agents that increase heart rate [catecholamines (dopamine/dobutamine/epinephrine), hypovolemia, elevated temperature], and managing systemic hypotension in the transition period with vasopressors that will not increase pulmonary vascular resistance or heart rate [10]. In some centers, intravenous vasopressin is the standard of care vasopressor in the transitional period due to its favorable properties on the pulmonary vascular bed. In addition, the impact of increased left ventricular afterload shifts the pressure volume loop favorably toward increased end-systolic volume; hence, higher stroke volume [10]. Clinicians should also be cautious when rewarming IDM infants undergoing therapeutic hypothermia for hypoxic-ischemic encephalopathy due to the consequential increase in heart rate. Intravenous Esmolol infusions (50–100 mcg/kg/min) followed by oral propranolol (3–4 mg/kg/day) have been used to improve cardiac function and LV outflow tract obstruction [11]. Of note, Esmolol should only be used in patients with a stable blood pressure (BP) and without active PH due to the negative effects on the pulmonary vascular bed. Esmolol is also ultra-short acting and allows for rapid titration and disappearance of β-blockade effect following discontinuation in the event of deleterious cardiac haemodynamic effects. It is important to consider the implications of β-blockade. Adverse cardiac effects include precipitation or worsening of decompensated heart failure (especially in the presence of pre-existing myocardial dysfunction) and bradyarrhythmia (due to its negative chronotropic effects). Another consideration is the adverse effects of β-blocker withdrawal, which is seen with the increased sympathetic effect of short-acting drugs such as propranolol. This may necessitate the need for a weaning period before cessation.

Other considerations for the general management of IDM include management of resistant hypoglycemic (potentially compounded by using a β-blocker), polycythaemia-associated hyperviscosity [12], and potential cardiac instability related to electrolyte imbalances (particularly hypocalcaemia and hypomagnesemia). Follow-up echocardiogram at 3–6 months post-discharge should be performed to show resolution of the hypertrophied intraventricular septum and free walls if affected. Any ongoing HCM, or where there is evidence of additional anatomic heart defects, should be investigated to exclude any genetic/familial cause, such as inborn errors of metabolism, malformation syndromes, neuromuscular disease, or sarcomeric/other cardiac genetic abnormalities [13]. Longer-term effects of exposure to *in utero* hyperglycemia and hyperinsulinism are unclear. Predisposition to atherosclerosis, the development of vascular disease, and a four-fold increased risk of metabolic syndrome in childhood have all been described in IDM [14, 15]. It is also unclear whether the IDM-associated hypertrophy leads to decreased myocardial endowment and the potential for impaired ventricle development. A more detailed follow-up may be required for these infants.

## Twin-to-twin transfusion syndrome recipient twins

Differential placental transfusion as seen in TTTS may also result in cardiac hypertrophy and a cardiomyopathy-type picture in recipient twins. TTTS is a fetal diagnosis with graded severity based on the significance of vascular anastomoses in the shared placenta between monochorionic diamniotic twin pairs. It is relatively rare but carries significant morbidity. Around 3% of live births are twins, of which 30% have monochorionic (MC) placentas, and 10–15% of these will develop TTTS [16–18]. Pre- and postnatal morbidity and mortality are increased, compounded by the high rates of premature birth (on average at 28–30 weeks’ gestation), with survival rates for both twins ranging only around only 50–65% and rates of long-term neurologic impairment in survivors at 10–20% [16, 19].

### Case 2

**Fig. 4:**
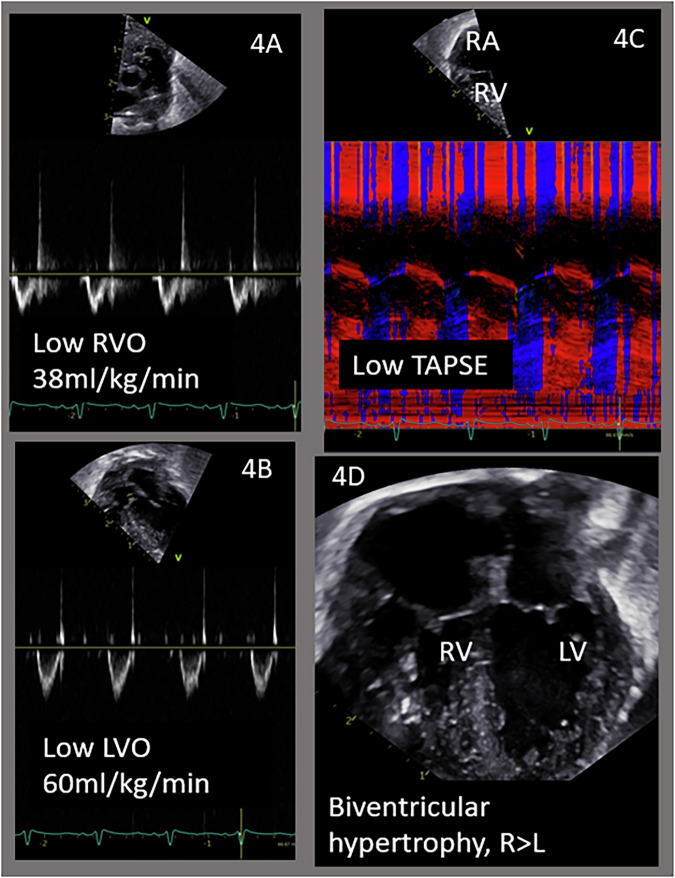
TTTS recipient twin case, selected echocardiography clips. **A** RVO- right ventricular output. **B** LVO- left ventricular output. **C** TAPSE- tricuspid annular plan systolic excursion. **D** ventricular hypertrophy. RV right ventricle, LV left ventricle.

This case of a TTTS recipient illustrates clearly the cardiovascular risk, but also successful close monitoring and intervention. A male monochorionic-diamniotic twin infant was born at 28w by cesarean section for progressing preterm labor in the setting of newly diagnosed TTTS stage IV. The baby was intubated at delivery, given surfactant, and received mechanical ventilation. He received a screening targeted neonatal echocardiogram (TNE) on postnatal day 1. At the time of assessment, he was on low ventilator settings and 0.21 FiO_2_, had a normal arterial lactate and pH, and normal BP for age. TNE evaluation revealed biventricular cardiac systolic dysfunction [LV markers included Simpsons biplane ejection fraction (LV EF) 40%]; RV markers [tricuspid annular planar systolic excursion (TAPSE) 2.6 mm, fractional area change (FAC) 16%, RV tissue Doppler imaging (TDI) s’ 2 cm/s] and hypertrophy (Fig. 4). In addition, there was evidence of biventricular decreased cardiac output (LV output 60 ml/kg/min, RV output 38 ml/kg/min). There was a small atrial communication and a small PDA, both with left-to-right shunting. An intravenous infusion of dobutamine at 5 mcg/kg/min was started. On TNE reassessment 4 h later, his LV function had normalized (LVEF 65%) but there was persistent mildly reduction in RV function (TAPSE 4.2 mm, FAC 40%, RV TDI s’ 3.5 cm/s), and improving but still low cardiac output (LV output 121 ml/kg/min and RV output 40 ml/kg/min). Low-dose epinephrine at 0.03 mcg/kg/min was added for further inotropic support, which led to normalization of ventricular function and outputs. He was successfully weaned off all therapies by postnatal day 5 and had no deleterious consequences.

#### Discussion

Placental vascular anastomoses (venous, arterial, and arterial-venous) occur in all MC pregnancies with shared blood flow between twins, but TTTS pathology develops when a net imbalance in blood flow occurs through significant arterial-venous connections. Diagnosis and prognostication are based on Quintero staging [20]. There are now additional staging systems that follow the expected cardiovascular progression of disease, but these aren’t yet in widespread use [21–23]. Fetal laser photocoagulation of placental anastomoses is now considered the gold standard approach to management in Quintero stage II+ if diagnosed prior to 26 weeks [18–24].

The unbalanced net flow between TTTS twins creates strikingly altered cardiac loading conditions and explains much of the morbidity facing the recipient twin. Donor twins typically compensate for their decreased cardiac preload and are less likely to present with clinically apparent disease at birth. The recipient twins are less able to tolerate their increased cardiac volume loading, and the developing heart can progressively develop a hypertrophic cardiomyopathy-type phenotype. There is growing evidence that in addition to volume loading, vasoactive mediators (renin, angiotensin) which are passed to the recipient twin from the donor (a compensatory response of the donor’s relative hypovolemic state) also contribute to the worsened recipient loading conditions from the addition of increased afterload and the effects of systemic hypertension [25, 26].

**Fig. 5:**
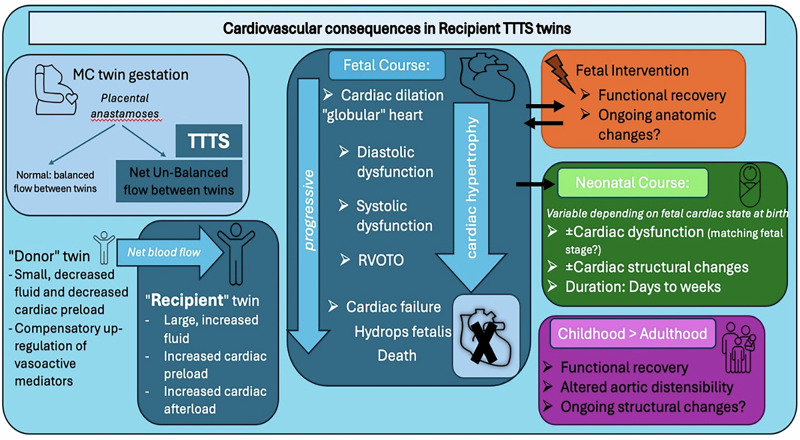
Cardiovascular consequences in twin-to-twin transfusion syndrome recipient twins. TTTS twin-to-twin transfusion syndrome, MC monochorionic, RVOTO right ventricular outflow tract obstruction.

The progression of cardiovascular disease in recipient twins seems to follow a somewhat predictable course (Fig. 5), although onset is variable and rapid worsening can occur [27, 28]. Accommodation of increased volume reliably causes changes in cardiac architecture, and early recipient twin disease can cause right-sided ventricular dilatation and more “globular” shaped hearts with increased cardiothoracic ratios [28, 29]. Next, subtle changes in diastolic function in both ventricles can occur, evidenced by worsened RV strain, prolonged isovolumetric relaxation time (IVRT), and increased ventricular filling pressures [28, 30, 31]. Systolic dysfunction follows, with a likely oversized contribution from the RV, with altered myocardial performance indices, shortening fractions, and new regurgitant jets [28, 32, 33]. If volume loading continues unchecked, then these cardiac consequences will progress, hypertrophy develops and worsens, and forward cardiac output falls, leading to diminished RV outflow and functional outflow tract obstruction in some cases, and in others progressive cardiac failure, hydrops fetalis, and death [21, 23].

While there are many fetal TTTS cardiovascular studies, detailed *postnatal* echocardiography assessments of recipient twins are few [34, 35]. However, even outside of the pathologic *in utero* environment, neonates affected by TTTS are at increased risk of morbidity and mortality compared to unaffected MC twins [16, 35]. This is likely multifactorial, but cardiovascular disease must certainly contribute. For instance, there is an increased incidence of congenital heart disease in surviving recipient twins even after ablative therapy was successfully performed *in utero*. The predominant congenital heart disease phenotype, RV outflow and functional outflow tract obstruction spectrum, fits their antenatal physiology [29, 34]. There are also data that show altered postnatal arterial wall stiffness in TTTS recipients and donors, particularly for those who did not receive laser ablative therapy, which further suggests fetal loading conditions influence long-term cardiovascular development and structure [36].

What little we know of cardiac function in neonates’ post-TTTS suggests that dysfunction, when present, normalizes with time; however, the course and predictors are unclear. One early study that examined recipient TTTS fetuses’ pre- and post-amnioreduction, where all fetuses had some degree of persistent cardiac dysfunction and hypertrophy post-intervention, found nearly half of the neonates displayed cardiac dysfunction at birth that normalized in most over several months [37]. Now, most patients are treated by fetal laser ablation, not amnioreduction, and after post successful procedure, many recipient TTTS fetuses normalize cardiac functional metrics [27, 33]. This would suggest that the subsequent cardiac normal function will be normal as well. However, one of the few recent studies that examined the post-natal transition period in TTTS showed persistent cardiac hypertrophy *and* significantly reduced markers of right-sided cardiac function in the second postnatal week in recipients who underwent successful laser photocoagulation *in utero*, compared to donors and compared to age-matched singletons [38]. The donor cohort demonstrated systemic artery (aortic) stiffness but preserved cardiac function. Putatively, vascular programming in monozygotic twins with intertwin transfusion may be altered but not abolished by intrauterine therapy to resemble that seen in dichorionic twins.

The stressors of transition to postnatal life, particularly abruptly increased afterload with the loss of the placenta and the risk of persistent pulmonary pressure elevation in immature lungs, could be poorly tolerated by an already dilated, hypertrophied, or dysfunctional TTTS recipient heart. Which hearts are at the highest risk, and which prenatal echocardiographic indicators may best risk-stratify patients, is unknown. Recent consensus guidelines for TNE recommend that infants with TTTS, regardless of antenatal treatment with laser photocoagulation, should undergo standard TNE assessments to identify pulmonary or systemic hemodynamics, characterize loading conditions, and assess myocardial performance [5]. A judicious approach to care may include careful consideration of early postnatal echocardiography in TTTS recipients, and careful consideration of the need for supportive interventions to augment function and/or reduce afterload in the correct scenario. Further study is critically needed in this population to examine, over multiple time points, the natural history of the recipient TTTS neonate and what interventions may best suit their unique physiology.

## Left heart pathology associated with BPD

Preterm infants are at a higher risk of developing severe BPD. Approximately 15,000 infants are affected in the United States annually, the incidence being consistently high in the NICHD Neonatal Research Network and the Australian and New Zealand Neonatal Network over the last two decades [39, 40]. The current definitions are limited by the fact that disease severity assumes a singular lung phenotype and is exclusively classified based on respiratory support rather than underlying etiological contributors. Published guidelines for assessment and management of BPD are exclusively based on screening for chronic pulmonary arterial hypertension and assume a single underlying phenotype [41, 42]. Little consideration has been paid to the contribution of systemic hemodynamics [systemic artery (aortic) dynamics and/or abnormal LV function] towards pulmonary vascular disease. Experimental, pathophysiological, and clinical data have indicated the role of systemic hemodynamics (systemic afterload and LV dysfunction) in neonatal-pediatric lung disease [43, 44]. Transudation of fluid across the pulmonary capillary beds will affect pulmonary compliance, influencing respiratory support requirements [43–45]. Recognition of this BPD phenotype, originating in the aorta and/or the LV, in unison or sequentially, may refocus treatment strategies in a subset of infants with severe lung disease.

### Case: 3

**Fig. 6:**
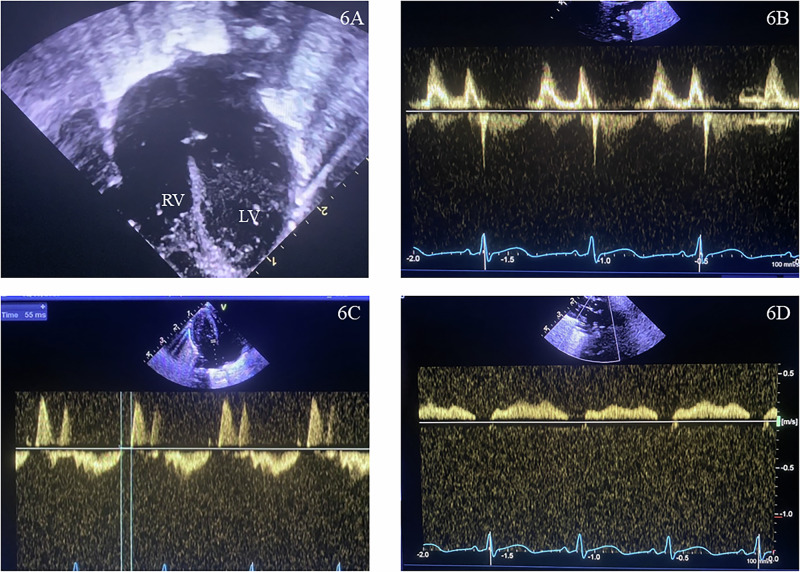
Pre-captopril echocardiographic information. **A** Left ventricular dilatation on 2D apical 4 chamber view. **B** Reversed trans-mitral E/A pulse Doppler. **C** Iso-volumic relaxation time. **D** Dampened pulmonary venous Doppler.

**Fig. 7:**
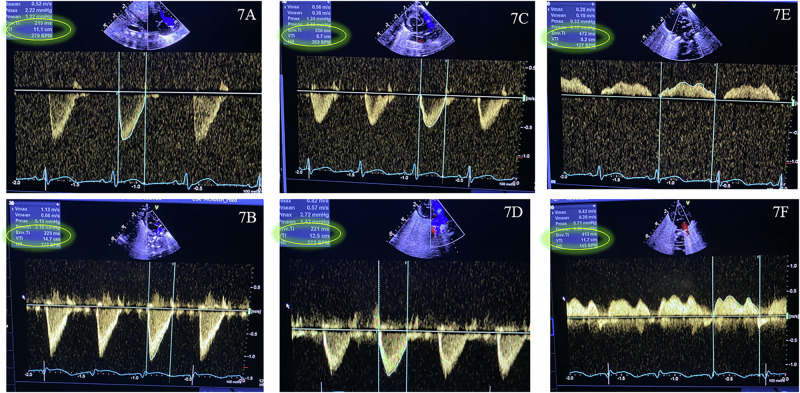
Evolution of cardiac indices with captopril. **A** and **B** Pre- and post-captopril left ventricular output velocity time integral. **C** and **D** Pre- and post-captopril right ventricular output velocity time integral. **E** and **F** Pre- and post-captopril pulmonary vein velocity time integral.

Table 1 describes a preterm infant who was mechanically ventilated from birth. Paracetamol was administered in the first week for a PDA, which resulted in successful closure. At 36 weeks post-menstrual age, he was still mechanically ventilated with FiO_2_ 0.6. Figure 6 summarizes echocardiography findings at 36 weeks, which noted LV dilatation, trans-mitral E/A ratio (>1), prolonged IVRT of 56 ms, and reduced pulmonary venous flow. No evidence of PH was noted (no tricuspid regurgitation or RV dilatation; time to peak velocity/right ventricular ejection time 0.3). On m-mode, the intima-media thickness in the abdominal aorta proximal to the celiac trunk was 990 µm (vs expected 674 ± 22 µm) with reduced pulsatility. His systemic systolic BP at 36 weeks gestation was >95th (87 mm Hg) and subsequently >99th centile for gestation from available charts (93 mm Hg) [46]. Captopril [angiotensin converting enzyme inhibitor (ACEi)] was administered initially at 0.1 mg/kg/dose every 8 h, gradually increasing to 0.5 mg/kg/dose over the next 5 weeks. Figure 7 depicts an increase in velocity time integrals for biventricular output and pulmonary venous flows in comparative echocardiograms before and after five weeks of captopril, which was accompanied by clinical improvements.

## Discussion

Infants with severe BPD have a higher incidence of systemic hypertension, which is associated with longer duration of need for respiratory support as well as greater risk of home oxygen [43, 46, 47]. Of concern, the approach to monitoring systemic hypertension is inconsistent and oftentimes infrequent. In a recent study, BP comparisons were made using gestation-specific 95th and 99th centiles [46]. In addition, elevated BP is often assumed to be artifactual when high, prompting repeat measurements until a normal value is achieved. In a recent study of preterm infants with severe BPD, aorta intima-media thickness and stiffness/impedance were measured by high-frequency vascular ultrasound, compared with preterm infants of equivalent gestational age but with no BPD and term infants [44]. Infants in the BPD cohort had increased thickness, impedance, and wall stiffness with reduced arterial compliance and beat-to-beat pulsatility. Lower distensibility and aortic compliance have been noted previously in low birthweight infants [48]. The additive effects of systemic hypertension and arterial stiffness in BPD infants may generate back-pressure changes in the left heart and pulmonary venous circulation. The consequential effects of increased left-atrial end-diastolic pressure (reflected in decreased pulmonary venous flow) on the immature lung initiate an edemagenic sequence (termed post-capillary pathophysiology). In terms of LV disease, a prospective echocardiographic study compared myocardial function in extremely preterm infants with severe BPD with preterm infants with no BPD [45]. Both diastolic and systolic function were adversely affected; however, trans-mitral E/A ratio, E-wave deceleration time, and IVRT time were impacted to a greater extent than LV output and fractional shortening. LV diastolic dysfunction is an important component of cPH pathophysiology in the Panama Classification of Pediatric Pulmonary Hypertensive Vascular Disease [49]. In addition, the European Society of Cardiology also separately categorizes patients with cPH with LV systolic/diastolic dysfunction [50]. These data parallel adult studies where cPH was seen in >60% of patients with LV systolic dysfunction and >80% of those with LV diastolic dysfunction [51, 52]. It is noteworthy that indices of LV diastolic function are rarely performed on routine screening echocardiography assessments in many centers [53].

In clinical situations where pulmonary vascular resistance mediated PH is not the cause (*functional*: systemic arterial stiffness/LV dysfunction, *flow based*: moderate-high volume PDA or atrial level shunt, or *anatomical*: pulmonary vein stenosis/mitral valve disease), non-judicious use of conventional pulmonary vasodilators [such as nitric oxide (iNO)/sildenafil] may be counter-productive. For instance, adult patients with PH secondary to mitral valve disease are excluded from clinical trials evaluating pulmonary vasodilator therapies [54]. In post-capillary cPH disease, knowledge of the underlying pathophysiology can guide therapeutic decision-making.

The renin-angiotensin-aldosterone system plays a role in arterial remodeling of the vasculature and the consequent arterial stiffness [55]. ACE-2 is a counter-regulatory enzyme of ACE [56]; specifically, during chronic hypoxemia (typical in severe BPD), ACE is upregulated while ACE-2 is downregulated, tipping the balance towards the pro-inflammatory (pro-fibrotic) effects, thereby promoting vascular remodeling [57, 58]. ACE inhibitors reset the balance between vasoconstrictors/ proliferators and vasodilators/anti-proliferators of the vascular wall, inhibit arterial remodeling and replacement of elastin fibers by collagen fibers [59–63], and improve endothelial function [64]. In adults, the mainstay of therapy for patients with cPH and accompanying LV dysfunction is not pulmonary vasodilator therapy; rather, systemic afterload reduction is employed to lower left atrial and pulmonary venous pressure. Clinical studies in infants with severe BPD support the therapeutic role of ACE inhibition [55, 65, 66]. In a study using cardiac catheterization in 13 preterm infants with severe BPD and cPH, administration of iNO demonstrated a net increase in wedge pressure in seven infants (58%), possibly related to co-existing impaired LV diastolic dysfunction [66]. Subsequently, in two premature infants with cPH and LV diastolic dysfunction, where respiratory support requirements and cardiac indices did not improve with diuretics and iNO, captopril resulted in significant clinical improvements and normalization of cardiac indices [65]. More recently, a similar therapeutic approach in a series of infants with severe BPD unresponsive to sildenafil and diuretic therapy resulted in significantly improved respiratory status and cardiac function and increased aortic pulsatility [55, 67, 68]. The prevalence of such infants amongst the broader BPD cohort is unclear, but greater surveillance using echocardiography and/or catheterization will inform true incidence. In our own experience over the last six years, the incidence is close to 10%, while a recent echocardiographic study over four years indicated this to be ~20% [69]. An earlier study using catheterization noted this physiology in 7/13 (58%) infants.

## Conclusions

Accurate hemodynamic assessment and management are critical in addressing the complex cardiovascular challenges faced by infants with conditions such as cardiomyopathy in IDM, TTTS, and BPD. These conditions exemplify the diverse and intricate hemodynamic alterations that can occur during the perinatal period, necessitating precise and tailored interventions. Overall, these clinical scenarios highlight the necessity for hemodynamic precision in neonatal care. By employing advanced diagnostic tools and adopting a physiology-driven approach, healthcare providers can enhance the management of these vulnerable populations, ultimately improving short- and long-term outcomes.

In conclusion, achieving hemodynamic precision in neonatal care is critical for effective management and improved outcomes in infants with complex cardiovascular conditions. Continued research and innovation in hemodynamic monitoring and therapeutic approaches are essential to bridge existing knowledge gaps and refine care strategies for these vulnerable populations. Supplementary Table 1 summarizes knowledge gaps and research priorities. Hemodynamic precision is paramount in guiding the care of neonates with complex cardiovascular conditions such as cardiomyopathy in IDM, TTTS, and BPD. Each of these conditions present unique hemodynamic challenges that require meticulous assessment and tailored interventions to optimize clinical outcomes. For IDM, precise measurement of cardiac function and structure is crucial in managing hypertrophic cardiomyopathy and preventing complications such as low cardiac output and PH. The variability in clinical presentation necessitates individualized hemodynamic evaluations to guide appropriate therapeutic interventions. In TTTS, the imbalanced blood flow between twins significantly alters cardiac loading conditions, particularly affecting the recipient twin. Serial TNE assessment is essential to understanding these changes and determining suitable interventions to alleviate cardiac strain and mitigate progression to heart failure or other complications. In the context of BPD, the interplay between systemic hypertension, arterial stiffness, and LV dysfunction requires precise hemodynamic monitoring to address the underlying cardiovascular contributions to pulmonary vascular disease. Interventions focused on afterload reduction may significantly improve cardiac and respiratory outcomes, underscoring the need for detailed systemic and pulmonary hemodynamic assessment.

Despite advancements in hemodynamic evaluation and management, several knowledge gaps remain. There is a need for standardized protocols to assess and interpret complex hemodynamic parameters in neonates accurately. Additionally, further research is required to understand the long-term cardiovascular consequences of these neonatal conditions and the impact of early hemodynamic interventions on future health outcomes. Identifying biomarkers and developing predictive models for adverse hemodynamic events could also enhance early detection and intervention strategies.

## Supplementary information

Supplementary Table 1

## References

[1] McNamara PJ, Abman SH, Levy PT. Reengagement with physiology in neonatal heart and lung care: a priority for training and practice. J Pediatr. 2024;268:113947. 10.1016/j.jpeds.2024.113947. 38336199 10.1016/j.jpeds.2024.113947

[2] Mirza H, Mandell EW, Kinsella JP, McNamara PJ, Abman SH. Pulmonary vascular phenotypes of prematurity: the path to precision medicine. J Pediatr. 2023;259:113444. 10.1016/j.jpeds.2023.113444. 37105409 10.1016/j.jpeds.2023.113444 PMC10524716

[3] Paauw ND, Stegeman R, de Vroede MAMJ, Termote JUM, Freund MW, Breur JMPJ. Neonatal cardiac hypertrophy: the role of hyperinsulinism—a review of literature. Eur J Pediatr. 2020;179:39–50. 10.1007/s00431-019-03521-6. 31840185 10.1007/s00431-019-03521-6 PMC6942572

[4] El-Ganzoury MM, El-Masry SA, El-Farrash RA, Anwar M, Abd Ellatife RZ. Infants of diabetic mothers: echocardiographic measurements and cord blood IGF-I and IGFBP-1. Pediatr Diabetes. 2020;13:189–96. 10.1111/j.1399-5448.2011.00811.x. 10.1111/j.1399-5448.2011.00811.x 21933314

[5] McNamara PJ, Jain A, El-Khuffash A, Giesinger R, Weisz D, Freud L, et al. Guidelines and recommendations for targeted neonatal echocardiography and cardiac point-of-care ultrasound in the neonatal intensive care unit: an update from the American Society of Echocardiography. J Am Soc Echocardiogr. 2024;37:171–215. 10.1016/j.echo.2023.11.016. 38309835 10.1016/j.echo.2023.11.016

[6] Elmekkawi SF, Mansour GM, Elsafty MSE, Hassanin AS, Laban M, Elsayed HM. Prediction of fetal hypertrophic cardiomyopathy in diabetic pregnancies compared with postnatal outcome. Clin Med Insights Womens Health. 2015. 10.4137/CMWH.S32825. 10.4137/CMWH.S32825 PMC4667560 26664250

[7] Kozák-Bárány A, Jokinen E, Kero P, Tuominen J, Rönnemaa T, Välimäki I. Impaired left ventricular diastolic function in newborn infants of mothers with pregestational or gestational diabetes with good glycemic control. Early Hum Dev. 2004;77:13–22. 10.1016/j.earlhumdev.2003.11.006. 15113627 10.1016/j.earlhumdev.2003.11.006

[8] Levy PT, Tissot C, Horsberg Eriksen B, Nestaas E, Rogerson S, McNamara PJ, et al. Application of neonatologist performed echocardiography in the assessment and management of neonatal heart failure unrelated to congenital heart disease. Pediatr Res. 2018;84:78–88. 10.1038/s41390-018-0075-z. 30072802 10.1038/s41390-018-0075-z PMC6257223

[9] Bolin EH, Spray BJ, Mourani PM, Porter C, Collins RT. Mortality among infants of diabetic mothers with hypertrophic cardiomyopathy. J Mater Fetal Neonatal Med. 2022;35:9893–9. 10.1080/14767058.2022.2066993. 10.1080/14767058.2022.2066993 35440277

[10] Boyd SM, Riley KL, Giesinger RE, McNamara PJ. Use of vasopressin in neonatal hypertrophic obstructive cardiomyopathy: case series. J Perinatol. 2021;41:126–33. 32951013 10.1038/s41372-020-00824-7

[11] Codazzi AC, Ippolito R, Novara C, Tondina E, Cerbo RM, Tzialla C. Hypertrophic cardiomyopathy in infant newborns of diabetic mother: a heterogeneous condition, the importance of anamnesis, physical examination and follow-up. Ital J Pediatr. 2021;47:197 10.1186/s13052-021-01145-x. 34593008 10.1186/s13052-021-01145-x PMC8485532

[12] Sehgal A, Francis JV. Haemodynamic consequences of polycythemia. J Perinatol. 2011;31:143–45. 21283081 10.1038/jp.2010.136

[13] Moak JP, Kaski JP. Hypertrophic cardiomyopathy in children. Heart. 2012;98:1044–54. 10.1136/heartjnl-2011-300531. 22591735 10.1136/heartjnl-2011-300531

[14] Kallem VR, Pandita A, Pillai A. Infant of diabetic mother: what one needs to know. J Mater Fetal Neonat Med. 2020;33:482–92. 10.1080/14767058.2018.1494710. 10.1080/14767058.2018.1494710 29947269

[15] Boney CM, Verma A, Tucker R, Vohr BR. Metabolic syndrome in childhood: association with birth weight, maternal obesity, and gestational diabetes mellitus. Pediatrics. 2005;115:290–6. 10.1542/peds.2004-1808. 10.1542/peds.2004-1808 15741354

[16] Sebire NJ, Snijders RJ, Hughes K, Sepulveda W, Nicolaides KH. The hidden mortality of monochorionic twin pregnancies. Br J Obstet Gynaecol. 1997;104:1203–7. 10.1111/j.1471-0528.1997.tb10948.x. 9333002 10.1111/j.1471-0528.1997.tb10948.x

[17] Osterman MJK, Hamilton BE, Martin JA, Driscoll AK, Valenzuela CP. Births: final data for 2021. Natl Vital Stat Rep. 2023;72:1–53. https://www.ncbi.nlm.nih.gov/pubmed/36723449. 36723449

[18] Shanahan MA, Bebbington MW. Monochorionic twins: TTTS, TAPS, and selective fetal growth restriction. Clin Obstet Gynecol. 2023;66:825–40. 10.1097/GRF.0000000000000821. 37910135 10.1097/GRF.0000000000000821

[19] Di Mascio D, Khalil A, D’Amico A, Buca D, Benedetti Panici P, Flacco ME, et al. Outcome of twin-twin transfusion syndrome according to Quintero stage of disease: systematic review and meta-analysis. Ultrasound Obstet Gynecol. 2020;56:811–20. 10.1002/uog.22054. 32330342 10.1002/uog.22054

[20] Quintero RA, Morales WJ, Allen MH, Bornick PW, Johnson PK, Kruger M. Staging of twin-twin transfusion syndrome. J Perinatol. 1999;19:550–5. 10.1038/sj.jp.7200292. 10645517 10.1038/sj.jp.7200292

[21] Rychik J, Tian Z, Bebbington M, Xu F, McCann M, Mann S, et al. The twin-twin transfusion syndrome: spectrum of cardiovascular abnormality and development of a cardiovascular score to assess severity of disease. Am J Obstet Gynecol. 2007;197:392.e1–8. 10.1016/j.ajog.2007.06.055. 10.1016/j.ajog.2007.06.055 17904973

[22] Stirnemann JJ, Nasr B, Proulx F, Essaoui M, Ville Y. Evaluation of the CHOP cardiovascular score as a prognostic predictor of outcome in twin-twin transfusion syndrome after laser coagulation of placental vessels in a prospective cohort. Ultrasound Obstet Gynecol. 2010;36:52–7. 10.1002/uog.7713. 20582931 10.1002/uog.7713

[23] Habli M, Michelfelder E, Cnota J, Wall D, Polzin W, Lewis D, et al. Prevalence and progression of recipient-twin cardiomyopathy in early-stage twin-twin transfusion syndrome. Ultrasound Obstet Gynecol. 2012;39:63–8. 10.1002/uog.10117. 21998013 10.1002/uog.10117

[24] Simpson LL. Twin-twin transfusion syndrome. Am J Obstet Gynecol. 2013;208:3–18. 10.1016/j.ajog.2012.10.880. 23200164 10.1016/j.ajog.2012.10.880

[25] Mahieu-Caputo D, Meulemans A, Martinovic J, Gubler M-C, Delezoide A-L, Muller F, et al. Paradoxic activation of the renin-angiotensin system in twin-twin transfusion syndrome: an explanation for cardiovascular disturbances in the recipient. Pediatr Res. 2005;58:685–8. 10.1203/01.PDR.0000180558.03164.E8. 16189193 10.1203/01.PDR.0000180558.03164.E8

[26] Galea P, Barigye O, Wee L, Jain V, Sullivan M, Fisk NM. The placenta contributes to activation of the renin angiotensin system in twin-twin transfusion syndrome. Placenta. 2008;29:734–42. 10.1016/j.placenta.2008.04.010. 18558429 10.1016/j.placenta.2008.04.010

[27] Eschbach SJ, Boons LS, Wolterbeek R, Middeldorp JM, Klumpera FJCM, Lopriore E, et al. Prediction of single fetal demise after laser therapy for twin-twin transfusion syndrome. Ultrasound Obstet Gynecol. 2016;47:356–62. 10.1002/uog.15753. 26395988 10.1002/uog.15753

[28] Noll ATR, Gijtenbeek M, Verweij E, Lewi L, Herling L, Haak MC. Cardiac adaptation and malformation in twin-twin transfusion syndrome and selective fetal growth restriction: a systematic review. Prenat Diagn. 2024;44:832–45. 10.1002/pd.6575. 38643403 10.1002/pd.6575

[29] Karatza AA, Wolfenden JL, Taylor MJ, Wee L, Fisk NM, Gardiner HM. Influence of twin-twin transfusion syndrome on fetal cardiovascular structure and function: prospective case-control study of 136 monochorionic twin pregnancies. Heart. 2002;88:271–7. 10.1136/heart.88.3.271. 12181221 10.1136/heart.88.3.271 PMC1767329

[30] Ortiz JU, Torres X, Eixarch E, Bennasar M, Cruz-Lemini M, Gómez O, et al. Differential changes in myocardial performance index and its time intervals in donors and recipients of twin-to-twin transfusion syndrome before and after laser therapy. Fetal Diagn Ther. 2018;44:305–10. 10.1159/000485380. 29353282 10.1159/000485380

[31] Raboisson MJ, Fouron JC, Lamoureux J, Leduc L, Grignon A, Proulx F, et al. Early intertwin differences in myocardial performance during the twin-to-twin transfusion syndrome. Circulation. 2004;110:3043–8. 10.1161/01.CIR.0000146896.20317.59. 15520320 10.1161/01.CIR.0000146896.20317.59

[32] Delabaere A, Wavrant S, Codsi E, Fouron JC, Raboisson MJ, Audibert F. Fetal Doppler in monochorionic pregnancies complicated by twin-to-twin transfusion syndrome and selective in utero growth restriction. Eur J Obstet Gynecol Reprod Biol. 2023;286:28–34. 10.1016/j.ejogrb.2023.04.006. 37182292 10.1016/j.ejogrb.2023.04.006

[33] Leszczynska K, Meyer-Szary J, Chojnicki M, Haponiuk I, Preis K, Stefanska K, et al. Assessment of cardiac function in donor and recipient fetuses during a 7-day follow-up after selective laser photocoagulation of communicating vessels due to TTTS. Ginekol Pol. 2019;90:189–94. 10.5603/GP.2019.0034. 31059111 10.5603/GP.2019.0034

[34] Herberg U, Gross W, Bartmann P, Banek CS, Hecher K, Breuer J. Long term cardiac follow up of severe twin to twin transfusion syndrome after intrauterine laser coagulation. Heart. 2006;92:95–100. 10.1136/hrt.2004.057497. 15814592 10.1136/hrt.2004.057497 PMC1860975

[35] Wagner S, Repke JT, Ural SH. Overview and long-term outcomes of patients born with twin-to-twin transfusion syndrome. Rev Obstet Gynecol. 2013;6:149–54. https://www.ncbi.nlm.nih.gov/pubmed/24826204. 24826204 PMC4002191

[36] Gardiner HM, Taylor MJ, Karatza A, Vanderheyden T, Huber A, Greenwald SE, et al. Twin-twin transfusion syndrome: the influence of intrauterine laser photocoagulation on arterial distensibility in childhood. Circulation. 2003;107:1906–11. 10.1161/01.CIR.0000060543.64250.80. 12665487 10.1161/01.CIR.0000060543.64250.80

[37] Fesslova V, Villa L, Nava S, Mosca F, Nicolini U. Fetal and neonatal echocardiographic findings in twin-twin transfusion syndrome. Am J Obstet Gynecol. 1998;179:1056–62. 10.1016/s0002-9378(98)70215-7. 9790398 10.1016/s0002-9378(98)70215-7

[38] Ting E, Teoh M, Sehgal A. Differential postnatal cardiovascular course of donor-recipient twins and associated pathophysiology-a cohort study. Am J Physiol Heart Circ Physiol. 2024;327:H1400–H1405. 10.1152/ajpheart.00656.2024. 39453436 10.1152/ajpheart.00656.2024

[39] Jensen EA, Schmidt B. Epidemiology of bronchopulmonary dysplasia. Birth Defects Res A Clin Mol Teratol. 2014;100:145–57. 24639412 10.1002/bdra.23235 PMC8604158

[40] Stoll BJ, Hansen NI, Bell EF, Walsh MC, Carlo WA, Shankaran S, et al. Eunice Kennedy Shriver National Institute of Child Health and Human Development Neonatal Research Network. Trends in care practices, morbidity, and mortality of extremely preterm neonates, 1993–2012. JAMA. 2015;314:1039–51. 26348753 10.1001/jama.2015.10244 PMC4787615

[41] Levy PT, Jain A, Nawaytou H, Teitel D, Keller R, Fineman J. for the Pediatric Pulmonary Hypertension Network (PPHNet) et al. Risk assessment and monitoring of chronic pulmonary hypertension in premature infants. J Pediatr. 2020;217:199–209. 31735418 10.1016/j.jpeds.2019.10.034

[42] Revanna GK, Kunjunju A, Sehgal A. Bronchopulmonary dysplasia associated pulmonary hypertension: making the best use of bedside echocardiography. Prog Pediatr Cardiol. 2017;46:39–43.

[43] Sehgal A, Steenhorst JJ, Mclennan DI, Merkus D, Ivy D, McNamara PJ. The left heart, systemic circulation and bronchopulmonary dysplasia: relevance to pathophysiology and therapeutics. J Pediatr. 2020;225:13–22. 32553872 10.1016/j.jpeds.2020.06.031

[44] Sehgal A, Malikiwi A, Paul E, Tan K, Menahem S. Systemic arterial stiffness in infants with bronchopulmonary dysplasia: potential cause of systemic hypertension. J Perinatol. 2016;36:564–9. 26914016 10.1038/jp.2016.10

[45] Sehgal A, Malikiwi A, Paul E, Tan K, Menahem S. A new look at Bronchopulmonary dysplasia: Post-capillary pathophysiology and cardiac dysfunction. Pulm Circ. 2016. 10.1086/688641. 10.1086/688641 PMC5210072 28090292

[46] Dionne JM, Abitbol CL, Flynn JT. Hypertension in infancy: diagnosis, management and outcome. Pediatr Nephrol. 2012;27:17–32. 21258818 10.1007/s00467-010-1755-z

[47] Sehgal A, Elsayed K, Nugent M, Varma S. Sequelae associated with systemic hypertension in infants with severe bronchopulmonary dysplasia. J Perinatol. 2022;42:775–80. 35354941 10.1038/s41372-022-01372-y PMC9184283

[48] Sehgal A, Doctor T, Menahem S. Cardiac function and arterial biophysical properties in small for gestational age infants: postnatal manifestations of fetal programming. J Pediatr. 2013;163:1296–300. 23896189 10.1016/j.jpeds.2013.06.030

[49] Cerro MJ, Abman S, Diaz G, Freudenthal AH, Freudenthal F, Harikrishnan S, et al. A consensus approach to the classification of pediatric pulmonary hypertensive vascular disease: report from the PVRI Pediatric Taskforce, Panama 2011. Pulm Circ. 2011;1:286–98. 21874158 10.4103/2045-8932.83456 PMC3161725

[50] Gali N, Hoeper MM, Humbert M. Guidelines for the diagnosis and treatment of pulmonary hypertension. Eur Heart J. 2009;30:2493–537. 19713419 10.1093/eurheartj/ehp297

[51] Lam CSP, Roger VL, Rodeheffer RJ, Borlaug BA, Enders FT, Redfield MM. Pulmonary hypertension in heart failure with preserved ejection fraction: a community based study. J Am Coll Cardiol. 2009;52:1119–26. 10.1016/j.jacc.2008.11.051 PMC2736110 19324256

[52] Walls MC, Cimino N, Bolling SF, Bach DS. Persistent pulmonary hypertension after mitral valve surgery: does surgical procedure affect outcome? J Heart Valve Dis. 2008;17:1–9. 18365562

[53] Baczynski M, Bell EF, Finan E, McNamara PJ, Jain A. Survey of practices in relation to chronic pulmonary hypertension in neonates in the Canadian Neonatal Network and the National Institute of Child Health and Human Development Neonatal Research Network. Pulm Circ. 2020;10:2045894020937126. 10.1177/2045894020937126. 32728420 10.1177/2045894020937126 PMC7366415

[54] Kiefer TL, Bashore TM. Pulmonary hypertension related to left-sided cardiac pathology. Pulm Med. 2011;2011:381787. 21660234 10.1155/2011/381787 PMC3109401

[55] Sehgal A, Krishnamurthy MB, Clark M, Menahem S. ACE inhibition for severe bronchopulmonary dysplasia-an approach based on physiology. Physiol Rep. 2018;6:e13821. 10.14814/phy2.13821. 30187692 10.14814/phy2.13821 PMC6125606

[56] Cheng XM, Hu YY, Yang T, Wu N, Wang XN. Reactive oxygen species and oxidative stress in vascular-related diseases. Oxid Med Cell Longev. 2022;2022:7906091. 10.1155/2022/7906091 PMC9001081 35419169

[57] Kattoor AJ, Pothineni NVK, Palagiri D, Mehta JL. Oxidative stress in atherosclerosis. Curr Atheroscler Rep. 2017;19:42. 28921056 10.1007/s11883-017-0678-6

[58] Ruan Y, Jiang S, Musayeva A, Gericke A. Oxidative stress and vascular dysfunction in the retina: therapeutic strategies. Antioxidants. 2020. 10.3390/antiox9080761. 10.3390/antiox9080761 PMC7465265 32824523

[59] Sehgal A, Alexander B, Morrison J, South AM. Fetal growth restriction and hypertension in the offspring: mechanistic links and therapeutic directions. J Pediatr. 2020;224:115–23. 32450071 10.1016/j.jpeds.2020.05.028 PMC8086836

[60] Wagenaar GT, Laghmani el H, Fidder M, Sengers RM, de Visser YP, de Vries L, et al. Agonists of Mas oncogene and angiotensin II type 2 receptors attenuate cardiopulmonary disease in rats with neonatal hyperoxia-induced lung injury. Am J Physiol Lung Cell Mol Physiol. 2013;305:L341–51. 23812633 10.1152/ajplung.00360.2012 PMC3763032

[61] South AM, Shaltout HA, Washburn LK, Hendricks AS, Diz DI, Chappell MC. Fetal programming and the angiotensin-(1-7) axis: a review of the experimental and clinical data. Clin Sci. 2019;133:55–74. 10.1042/CS20171550 PMC6716381 30622158

[62] South AM, Diz DI, Chappell MC. COVID-19, ACE2, and the cardiovascular consequences. Am J Physiol Heart Circ Physiol. 2020;318:H1084–H1090. 32228252 10.1152/ajpheart.00217.2020 PMC7191628

[63] Morrell NW, Atochina EN, Morris KG, Danilov SM, Stenmark KR. Angiotensin converting enzyme expression is increased in small pulmonary arteries of rats with hypoxia-induced pulmonary hypertension. J Clin Invest. 1995;96:1823–33. 7560074 10.1172/JCI118228 PMC185819

[64] Neutel JM. Effect of the renin–angiotensin system on the vessel wall: using ACE inhibition to improve endothelial function. J Hum Hypertens. 2004;18:599–606. 15190263 10.1038/sj.jhh.1001714

[65] Mourani PM, Ivy DD, Rosenberg AA, Fagan TE, Abman SH. Left ventricular diastolic dysfunction in bronchopulmonary dysplasia. J Pediatr. 2008;152:291–3. 18206706 10.1016/j.jpeds.2007.11.006 PMC2259289

[66] Khemani E, McElhinney DB, Rhein L, Andraade O, Lacro RV, Thomas KC, et al. Pulmonary artery hypertension in formerly premature infants with bronchopulmonary dysplasia: clinical features and outcomes in the surfactant era. Pediatrics. 2007;120:1260–9. 18055675 10.1542/peds.2007-0971

[67] Reyes-Hernandez ME, Bischoff AR, Giesinger RE, Rios DR, Stanford AH, McNamara PJ. Echocardiography Assessment of Left Ventricular Function in Extremely Preterm Infants, Born at Less Than 28 Weeks’ Gestation, With Bronchopulmonary Dysplasia and Systemic Hypertension. J Am Soc Echocardiogr. 2024;37:237–47. 37619910 10.1016/j.echo.2023.08.013

[68] Stanford AH, Reyes M, Rios DR, Giesinger RE, Jetton JG, Bischoff AR, et al. Safety, Feasibility, and Impact of Enalapril on Cardiorespiratory Physiology and Health in Preterm Infants with Systemic Hypertension and Left Ventricular Diastolic Dysfunction. J Clin Med. 2021;10:4519. 10.3390/jcm10194519. 34640535 10.3390/jcm10194519 PMC8509219

[69] Gopagondanahalli KR, Ng WD, Tan JM, Rajadurai VS, Sundararaghavan S, Ang WL, et al. Clinical and Echocardiographic Response in Bronchopulmonary Dysplasia Associated Pulmonary Hypertension with Sildenafil Therapy in Extreme Preterm Infants. Perinatal Society of Australia and New Zealand Annual Congress 2024, Christchurch, New Zealand. Abstract 32.

