# Supplementary material for: Diagnostic and therapeutic precision in cardiovascular diseases in the neonatal intensive care
Source: J Perinatol. 2025 May 10;45(7):888–99. doi: 10.1038/s41372-025-02317-x (PMC12316595; doi:10.1038/s41372-025-02317-x)
Supplement: Supplementary file 1 — Supplementary Table 1 [file 41372_2025_2317_MOESM1_ESM.docx]

Supplementary Table 1: Knowledge gaps and research priorities

| IDM   1. Investigation of foetal correlates of postnatal hemodynamic outcomes 2. For IDM cohorts with cardiovascular impairments, standardizing echocardiographic surveillance and instituting physiologically appropriate cardio-supportive therapies. 3. Explore mechanistic links between IDM and future risks of cardio-metabolic syndromes. 4. Assessment of cardiovascular outcomes of affected infants into later childhood and early adulthood.   TTTS   1. Echocardiography stratification during prenatal period to identify high-risk cohorts within the pregnancies managed by laser photocoagulation. 2. Systematic follow-up of recipient hearts as they have dilated and hypertrophied cardiac anatomy with impaired function during neonatal period. 3. Donor cohorts have higher BP and stiffer vessels, possibly in keeping with the ‘developmental origins of human adult diseases’ postulations. So, BP tracking, avoiding catch-up phenotype and avoidance of excessive weight gain, in an attempt to reduce future risk of metabolic syndromes, should be studied. 4. Assessment of cardiovascular outcomes of affected infants into later childhood and early adulthood.   BPD associated PH   1. Enhanced surveillance of blood pressure patterns in term corrected preterm infants with use of gestation specific BP centiles (50^th^, 95^th^ and 99^th^) rather than mean BP. 2. Equal emphasis on ‘systemic’ haemodynamics and surveillance for post- 3. capillary phenotypes in screening echocardiograms for infants with severe BPD. 4. Investigation of the biological determinants of post-capillary phenotypes (vascular compliance, renin-angiotensin system, left ventricular development) 5. Multicentre data collection to document incidence of systemic hypertension. 6. Given the experimental and preliminary clinical evidence of Angiotensin Converting Enzyme inhibitors in improving pulmonary symptomatology in post-capillary PH, randomized controlled trials to compare ACE inhibition with other anti-hypertensives (calcium channel/ꞵ-blockers) could be explored. |
| --- |

IDM-infant of diabetic mothers, BPD-bronchopulmonary dysplasia, TTTS-twin to twin transfusion syndrome, BP-blood pressure, PH-pulmonary hypertension.
